# Formation of functional areas in the cerebral cortex is disrupted in a mouse model of autism spectrum disorder

**DOI:** 10.1186/s13064-015-0033-y

**Published:** 2015-04-03

**Authors:** Laura R Fenlon, Sha Liu, Ilan Gobius, Nyoman D Kurniawan, Skyle Murphy, Randal X Moldrich, Linda J Richards

**Affiliations:** Queensland Brain Institute, The University of Queensland, Building 79, St Lucia Campus, Brisbane, QLD 4072 Australia; Centre for Advanced Imaging, The University of Queensland, Brisbane, QLD 4072 Australia; The School of Biomedical Sciences, The University of Queensland, St Lucia Campus, Brisbane, QLD 4072 Australia; Current address: UQ Centre for Clinical Research, The University of Queensland, Royal Brisbane & Women’s Hospital Campus, Brisbane, Queensland 4029 Australia

**Keywords:** Autism spectrum disorders (ASD), BTBR mice, Agenesis of the corpus callosum, Cortical area patterning, Diffusion imaging

## Abstract

**Background:**

Autism spectrum disorders (ASD) are a group of poorly understood behavioural disorders, which have increased in prevalence in the past two decades. Animal models offer the opportunity to understand the biological basis of these disorders. Studies comparing different mouse strains have identified the inbred BTBR T + tf/J (BTBR) strain as a mouse model of ASD based on its anti-social and repetitive behaviours. Adult BTBR mice have complete agenesis of the corpus callosum, reduced cortical thickness and changes in early neurogenesis. However, little is known about the development or ultimate organisation of cortical areas devoted to specific sensory and motor functions in these mice that may also contribute to their behavioural phenotype.

**Results:**

In this study, we performed diffusion tensor imaging and tractography, together with histological analyses to investigate the emergence of functional areas in the cerebral cortex and their connections in BTBR mice and age-matched C57Bl/6 control mice. We found evidence that neither the anterior commissure nor the hippocampal commissure compensate for the loss of callosal connections, indicating that no interhemispheric neocortical connectivity is present in BTBR mice. We also found that both the primary visual and somatosensory cortical areas are shifted medially in BTBR mice compared to controls and that cortical thickness is differentially altered in BTBR mice between cortical areas and throughout development.

**Conclusions:**

We demonstrate that interhemispheric connectivity and cortical area formation are altered in an age- and region-specific manner in BTBR mice, which may contribute to the behavioural deficits previously observed in this strain. Some of these developmental patterns of change are also present in human ASD patients, and elucidating the aetiology driving cortical changes in BTBR mice may therefore help to increase our understanding of this disorder.

**Electronic supplementary material:**

The online version of this article (doi:10.1186/s13064-015-0033-y) contains supplementary material, which is available to authorized users.

## Background

Autism spectrum disorders (ASD) represent a group of behaviourally defined neurodevelopmental disorders associated with disruptions of social, cognitive and/or motor behaviours. Increased understanding and acceptance of ASD in recent clinical practice indicates that the prevalence of ASD has reached a median rate of 13/10,000 in young children, or even 1% in certain populations, with a male/female ratio of 4.4:1 [1]. However, although it is suggested that the complex impairments observed in ASD patients are caused by a number of genetic and environmental factors, as well as immunological and metabolic status, the neurological aetiology of ASD remains unclear [2-9]. Previous neuroimaging studies have revealed several conserved anatomical alterations in autistic patients that may be relevant to their behavioural abnormalities [10]. These include increased total brain volume, decreased grey matter volume in the amygdala-hippocampal complex, a thinner corpus callosum and malformations of the ventricular system [11-13].

Although autism is a human disorder, some of its characteristic behavioural phenotypes can be modelled in animals, facilitating experimental investigations of the anatomical and functional abnormalities that may be associated with ASD [14-18]. BTBR T + tf/J (BTBR) is an inbred mouse strain that displays robust behavioural phenotypes analogous to the three major diagnostic criteria of autism: aberrant reciprocal social interactions, deficits in social communication and repetitive stereotypic behaviours [14,19,20]. Neuroanatomical studies using magnetic resonance imaging (MRI) and diffusion tensor MRI have demonstrated that BTBR mice display anatomical abnormalities that include volume changes in the cerebral white matter and grey matter and disruptions in the major white matter tracts of the brain [16,17,21,22]. Most of these studies have focused on white matter malformations, revealing complete agenesis of the corpus callosum, thinning of the hippocampal commissure and the presence of ectopic interhemispheric connectivity above the third ventricle [16], subcortically through the posterior cerebrum [21] and excessively through the anterior commissure [17]. However, despite the ASD-like traits commonly exhibited by humans with agenesis of the corpus callosum [23], the behavioural trait of low sociability in mouse models does not appear to be related to the presence or size of this commissure [24,25], indicating that other developmental changes in the brain may be associated with this trait.

Recent data from genetic studies of human autistic patients have suggested that genes regulating the formation of the cortical areas specialised for the processing of sensory or motor information could be affected in ASD patients [26-28]. Our knowledge of how cortical areas are formed is primarily based on gene expression and mutational analyses in mice. However, whether BTBR mice display malformations in the formation of cortical sensory areas or their region-specific connections is unknown.

In this study, we investigated the development of cortical areas and neocortical interhemispheric connectivity in BTBR mice compared with C57Bl/6 mice. The inbred C57Bl/6 mouse strain is commonly used as a control for studies in BTBR mice as it displays high sociability and low repetitive self-grooming behaviours [19]. Diffusion MRI and tractography were used to examine interhemispheric connectivity and confirmed the complete absence of the corpus callosum and a thinner hippocampal commissure in the BTBR mice. However, neither the anterior commissure nor the hippocampal commissure appears to anatomically compensate for the loss of callosal connections in these mice. In addition, protein expression analyses and measurements were conducted to investigate the formation of sensory areas in the cerebral cortex and area-specific connections. We found that the thickness and size of the cortical areas is altered in a region- and age-dependent manner, and both the primary somatosensory (S1) and primary visual (V1) cortical areas are shifted medially in BTBR mice. These findings provide an increased understanding of developmental changes in the cortex of an animal model of ASD and add to our current knowledge of the pathophysiological processes that may potentially occur in this developmental disorder.

## Results

### BTBR mice display a severe neocortical interhemispheric disconnection

The anterior commissure, the hippocampal commissure and the corpus callosum are the three major commissural tracts present in the mouse and human forebrain. Although it is clear that BTBR mice have complete agenesis of the corpus callosum [22], the extent of neocortical disconnection through other commissures remains unclear. Results from previous investigations of telencephalic commissures in the BTBR mouse have been inconsistent, with some finding novel ectopic tracts connecting the cortical hemispheres [16,21], and others finding an increase in fibres crossing through the anterior commissure [17]. To examine these axon tracts and the degree of interhemispheric connectivity in three dimensions in mice, we performed diffusion MRI and tractography on adult BTBR and C57Bl/6 brains (*n* = 6 for each strain).

**Figure 1:**
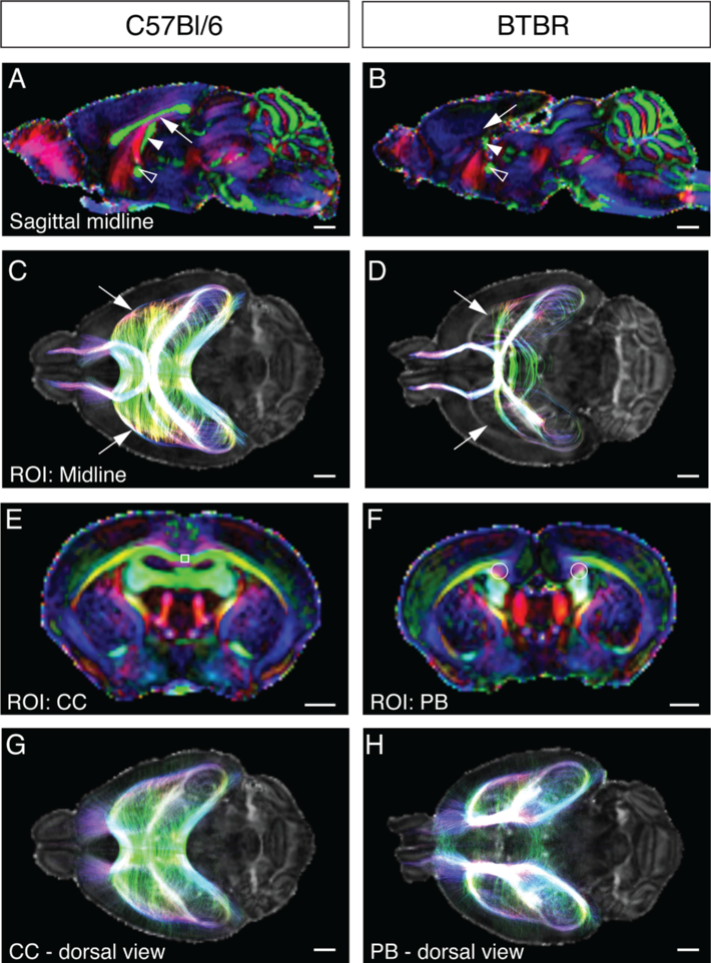
**Agenesis of the corpus callosum and Probst bundle formation in adult BTBR mice. (A, B)** Representative colour fractional anisotropy maps in a mid-sagittal plane of adult C57Bl/6 and BTBR mouse strains. Absence of the corpus callosum can be observed in BTBR mice (white arrow in (B)). A partial remnant of the hippocampal commissure is present in the BTBR mice (white arrowhead in (B)). The anterior commissure is preserved and is in a similar position in both mouse strains (open arrowheads in (A) and (B)). **(C, D)** Representative dorsal views of the tractography shows a large region of dispersed green streamlines connecting the two sides of the brain in C57Bl/6 mice (white arrows in (C)), whereas in BTBR mice this is absent (white arrows in (D)). In the coronal plane **(E, F)**, the corpus callosum is formed in C57Bl/6 mice, whereas Probst bundles are present on both sides of the midline in BTBR mice (white circles in (F)). All callosal streamlines passing through the corpus callosum at the midline (white box in (E)) are shown in **(G)**, and all streamlines passing through the Probst bundle in each hemisphere are shown in **(H)**. The fibres within the Probst bundles run rostrocaudally, connecting hippocampal and basal forebrain regions. Orientations for the colour fractional anisotropy maps: red, rostrocaudal; green, mediolateral; blue, dorsoventral. Scale bar = 1 mm. ROI = region of interest; CC = corpus callosum, PB = Probst bundles; BTBR = BTBR T + tf/J.

In the C57Bl/6 mice, the callosal axons constitute a long band of axons in the sagittal plane projecting across the midline (white arrow in Figure 1A). We first confirmed that the corpus callosum in BTBR mice is absent at the midline in the sagittal view (white arrow in Figure 1B). It has been demonstrated that in animals and humans with callosal malformations, callosally projecting axons aggregate on either side of the midline to form longitudinal axon bundles called Probst bundles [29-32]. In accordance with previous results [16,17,25,33], we demonstrated that Probst bundles are present on both sides of the midline in 100% of cases in coronal planes of BTBR brains (white circles in Figure 1F), but not in any of the C57Bl/6 brains (Figure 1E). By selecting a region of interest (ROI) in the corpus callosum of C57Bl/6 mice (white box in Figure 1E) we observed that streamlines generated through this ROI run mediolaterally, crossing the midline and connecting the left and right cortices (Figure 1G), whereas streamlines generated from ROIs of the Probst bundles (white circles in Figure 1F, with 10,000 streamlines generated from these ROIs) form longitudinal tracts, which project caudally towards the hippocampus or ventrally to the basal forebrain (Figure 1H), as previously described in other acallosal mouse strains [32,34].

**Figure 2:**
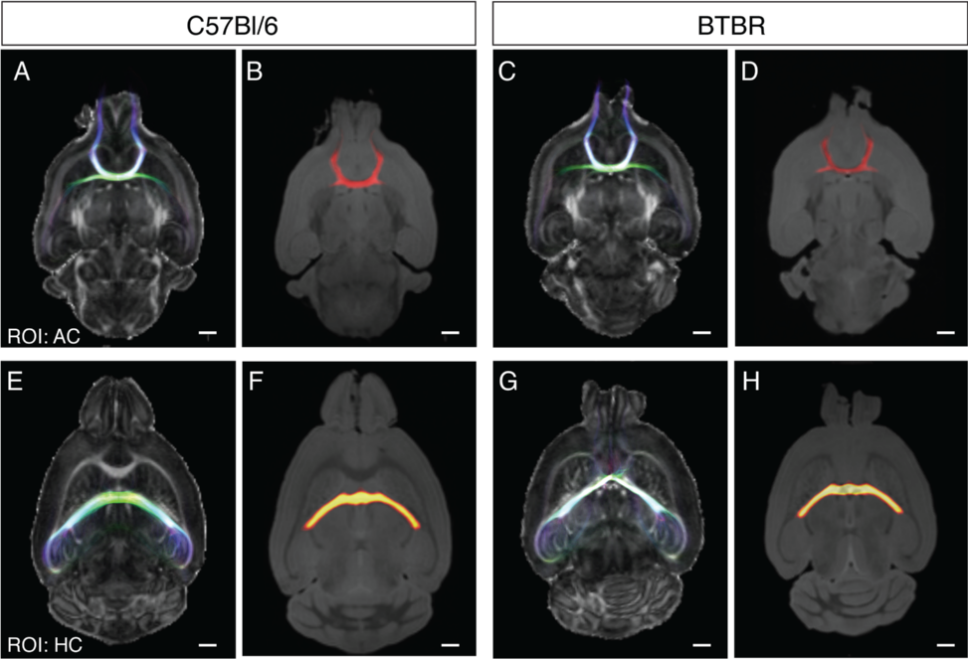
**Reconstruction of the anterior and hippocampal commissures and quantification of structural volumes.** Representative dorsal view of reconstructed fibre tract streamlines via the anterior commissure **(A, C)** and the hippocampal commissure **(E, G)** in C57Bl/6 **(A, E)** and BTBR **(C, G)** adult mouse brains. Streamlines were overlaid on fractional anisotropy maps. Structural volumes of the anterior commissure **(B, D)** and the hippocampal commissure **(F, H)** in C57Bl/6 **(B, F)** and BTBR **(D, H)** adult mouse brains derived from anatomical T_1_-weighted MRI images. Scale bar = 1 mm. ROI = region of interest; HC = hippocampal commissure, AC = anterior commissure.

Next, we investigated the degree of connectivity of the other traditional cortical commissures in BTBR mice, first confirming previous findings that the hippocampal commissure is present but greatly reduced in size in the BTBR strain (7,639 ± 1,015 streamlines in BTBR mice compared to 9,441 ± 100 streamlines in C57Bl/6 mice, *P* = 0.0072 Student’s *t*-test; white arrowhead in Figure 1B, and Figure 2E,G). However, contrary to previous reports [17], we found that the anterior commissure is preserved, but not enlarged (but rather reduced) in the BTBR strain (8,612 ± 548 streamlines in BTBR mice compared to 9,587 ± 409 streamlines in C57Bl/6 mice, *P* = 0.0065 Student’s *t*-test; open arrowhead in Figure 1A,B and Figure 2A,C). Similarly, by measuring the volume of each of these commissures from our 3D MRI data (normalised to the size of the brain; *n* = 6 for each strain; Figure 2B,D,F,H), we found that the volume of both the anterior (0.73 ± 0.02% in C57Bl/6 and 0.67 ± 0.01% in BTBR mice, *P* = 0.0008, Student’s *t*-test) and hippocampal (0.53 ± 0.03% in C57Bl/6 and 0.42 ± 0.02% in BTBR mice, *P* = 0.0001, Student’s *t*-test) commissures are significantly reduced in BTBR mice.

The lack of enlargement of the anterior and hippocampal commissures in the BTBR strain does not conclusively exclude the possibility of abnormal neocortical connectivity, as axons from cortical regions normally connected by the callosum could reroute through these commissures at the expense of the areas that they usually connect. To address whether specific functional cortical areas are connected via the anterior commissure or hippocampal commissure in BTBR mice, ROIs were placed in the bilateral motor, somatosensory and visual areas of the cortex in both C57Bl/6 and BTBR brains, and tractography was performed with inclusion ROIs in each commissure. Our results revealed that, within 10,000 streamlines generated through each commissure, there are no significant streamline connections between homologous interhemispheric areas via the anterior or hippocampal commissure in BTBR compared to C57Bl/6 mice. Together, these results show that neither the anterior commissure nor the hippocampal commissure compensates for the loss of callosal connections between functionally similar neocortical interhemispheric areas in BTBR mice.

Finally, we investigated the presence of ectopic interhemispheric connectivity in the BTBR strain by reconstructing all of the axons crossing the midline. We observed elaborate connections between the two hemispheres in C57Bl/6 mice, including between the bilateral olfactory bulbs, cortices and hippocampi (Figure 1C). However, contrary to previous reports showing ectopic neocortical commissures in the BTBR strain [16,21], we found that streamlines connecting the two cortices are absent in these mice (compare white arrows in Figure 1C,D), leaving only streamlines connecting the two olfactory bulbs via the anterior commissure and the hippocampi via the hippocampal commissure. Collectively, these results show that the neocortices of BTBR mice are not connected by the corpus callosum, through other forebrain commissures, nor via ectopic forebrain connections.

### The size and position of primary neocortical areas is altered in BTBR mice

**Figure 3:**
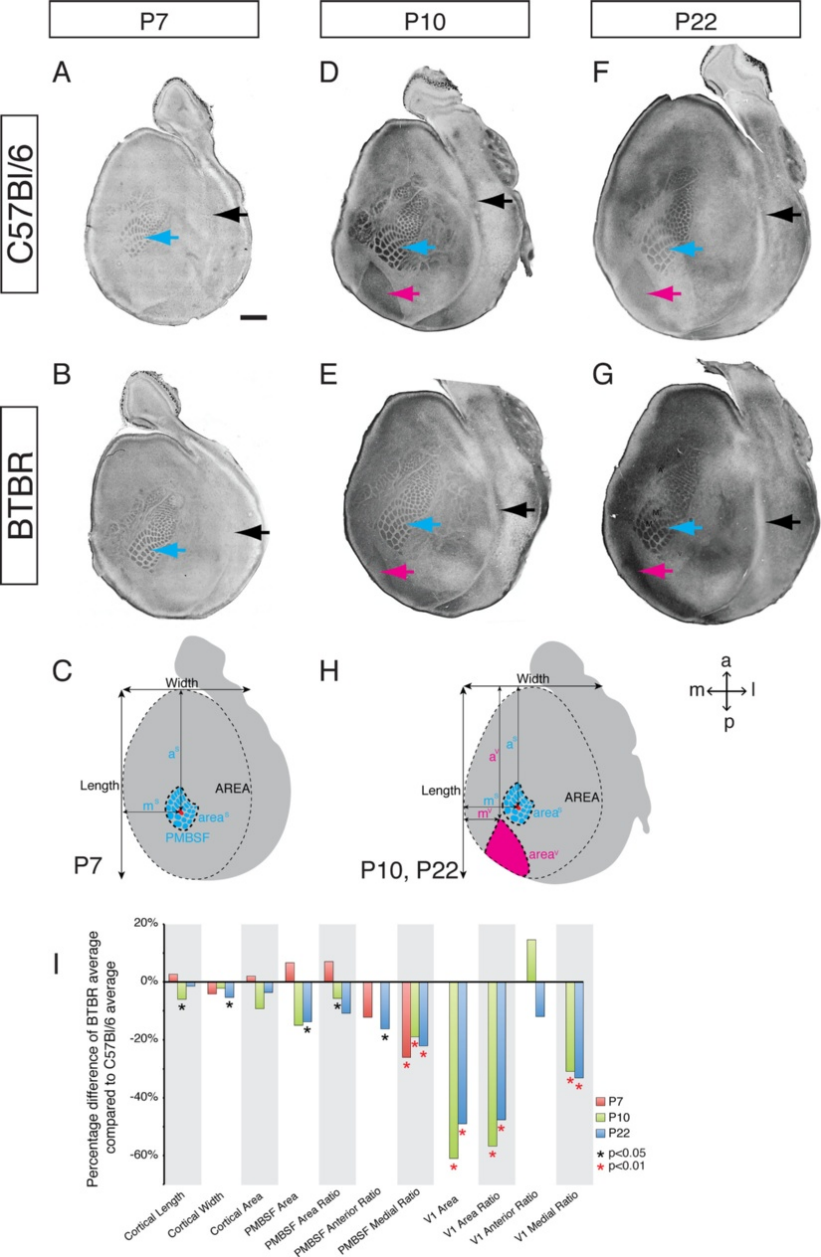
**Medially shifted somatosensory and visual areas and a reduction in primary visual area in developing BTBR mice.** Tangential sections through layer 4 of flattened cortices stained for anti v-Glut2 were obtained from P7 **(A, B)**, P10 **(D, E)** and P22 **(F, G)** C57Bl/6 **(A, D, F)** and BTBR **(B, E, G)** mice (*n* **≥** 6 for each age and strain). V-Glut2-positive PMBSF barrels in S1 at P7, P10 and P22 are indicated with blue arrows, and the v-Glut2-positive V1 is indicated by magenta arrows; the rhinal fissure (demarcating the border between neocortex and olfactory cortex) is indicated by black arrows. **(C, H)** Schematics of P7 **(C)** or P10 and P22 tangential sections **(H)** illustrate the cortical arealisation parameters that were measured in BTBR and C57Bl/6 mice. **(I)** Quantification of the cortical arealisation parameters in both strains, including total cortical length, width and area; total PMBSF area; PMBSF area normalised to total cortical area; PMBSF position relative to the anterior extent of the cortex; PMBSF position relative to the medial extent of the cortex; total V1 area; V1 area normalised to total cortical area; V1 position relative to the anterior extent of the cortex; and V1 position relative to the medial extent of the cortex. Measurements relating to V1 arealisation were only obtained at P10 and P22, as the V1 area is indistinct at P7 in tangential sections. Measurements were averaged for each strain and displayed as the percentage difference between BTBR and C57Bl/6 (100%). Orientations of tangential sections are indicated in the upper right corner of **(H)**: anterior/rostral to the top, posterior/caudal to the bottom, medial to the left and lateral to the right. Scale bar = 1 mm, BTBR = BTBR T + tf/J, PMBSF = posterior medial barrel subfield.

It is possible that the absence of neocortical interhemispheric connectivity in BTBR mice may have subsequent effects on the postnatal development of the neocortex, as callosal axons no longer innervate their normal targets [35,36]. Therefore, we next investigated whether the formation of neocortical areas, particularly in terms of their topographic organisation and relative positions along the medial-lateral and anterior-posterior axes, is altered in BTBR mice. As S1 (blue arrowheads in Figure 3) and V1 (magenta arrowheads in Figure 3) are the most readily identifiable primary neocortical areas in mice, we focused on the formation and position of these areas at different postnatal stages (P7, P10 and P22), using anti-v-Glut2 immunohistochemistry in tangential sections to visualise the axons terminals of glutamatergic neurons within layer 4 [37,38]. All cortical arealisation measurements were performed blind using de-identified images.

When we investigated the shape and number of individual barrels within S1, we observed no difference between BTBR and C57Bl/6 brains at any of the examined stages (blue arrows in Figure 3A,B,D,E,F,G; *n* ≤ 6 for each strain). We next analysed the spatial position of the posterior medial barrel subfield (PMBSF) with respect to its position in the anterior-posterior and medial-lateral axes of the cortex (schematics shown in Figure 3C,H), as the topography of this structure is highly conserved across different mouse strains [39]. Specifically, the spatial position of the PMBSF within the neocortex was determined by identifying the position of the third barrel in row C (C3 barrel; highlighted in red in Figure 3C,H). These measurements revealed that the C3 barrel is located in a more medial relative position in BTBR brains than in C57Bl/6 brains at all ages examined (Figure 3I and Additional file 1). Furthermore, the anterior-posterior position of the C3 barrel is relatively similar in both strains, except in P22 BTBR mice, where it is shifted significantly anteriorly (Figure 3I and Additional file 1).

Further measurements revealed that the total cortical area is similar in C57Bl/6 and BTBR mice at P10 and P22 (*n* = 8 for each strain), yet the length of the BTBR cortex is reduced at P10, and the width is reduced at P22. In addition, the relative size of the PMBSF is reduced at P10, and similarly, the relative size of the visual area is significantly reduced at both P10 and P22 (Figure 3I and Additional file 1), perhaps indicating fluctuations in the rate at which different cortical areas grow in BTBR brains compared to controls.

Together, our results indicate that V1 is reduced in size relative to the total cortical area in BTBR mice, which may impact the relative position of S1. Moreover, the relative positions of both the PMBSF and V1 are generally normal along the anterior-posterior axis at most postnatal ages but are significantly shifted towards the midline in BTBR mice at all stages of development, irrespective of changes in cortical size.

### BTBR mice display altered cortical thickness at different developmental stages that is not due to changes in the relative proportion of specific cortical layers

**Figure 4:**
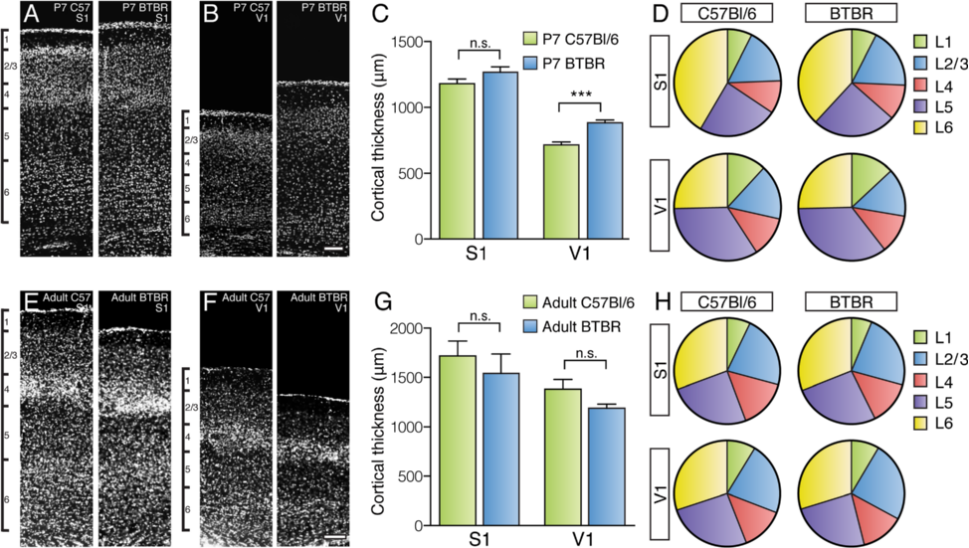
**Cortical thickness and layer proportion analysis in C57Bl/6 and BTBR mice at different developmental stages**. Coronal sections of C57Bl/6 and BTBR brains were stained with nuclear markers, and the thickness of the cortical plate, as well as its composite layers, was measured in P7 and adult animals. Qualitative **(A, B)** and quantitative **(C)** analysis reveals a significant increase in cortical thickness of V1, but not S1, at P7 (*n* = 7 animals for each strain, Student’s *t*-tests). However, the proportion of layers that comprise the cortical plate is not significantly different between strains (*n* = 7 animals for each strain; thickness of each layer as a percentage of total cortical thickness compared with Student’s *t*-tests; **(D)**). By adulthood, there is no significant difference in cortical thickness between the two strains, although there is a general trend towards a thinner cortex in the BTBR mouse **(E-G)**. The proportion of layers comprising the cortical plate is again unchanged in adulthood between the two strains (*n* = 6 animals for each strain; **(H)**). ***P < 0.001. Scale bar = 100 μm for (A) and (B) and 200 μm for (E) and (F). BTBR = BTBR T + tf/J, n.s. = not significant.

Given the observed changes in the size and position of cortical areas of BTBR mice, we next investigated whether BTBR brains also display alterations in the formation and lamination of the cortical plate. It has been reported that the proliferation, migration and apoptosis of cortical neurons may be altered in BTBR mice [40,41], which may subsequently influence the final distribution of neurons in different cortical layers. To examine this further, we first assessed the absolute thickness of the cortex (in the coronal plane), and the relative proportion of cortical layers in early postnatal and adult animals, in two cortical areas (S1 and V1) using nuclear staining analysis (Figure 4; Additional file 2). We found that the thickness of V1 in BTBR brains is significantly increased compared to that of C57Bl/6 mice at P7 (*P* = 0.0002, Student’s *t*-test) whereas that of S1 is unchanged (Figure 4A,B,C; *n* = 7 for both strains). Interestingly, we found that this trend is altered in adult animals, such that the thickness of S1 and V1 is not significantly different between the two strains, although there is a trend towards a reduced thickness in the BTBR mouse (Figure 4E,F,G; *n* = 6 for both strains). The relative thickness of the layers that make up the cortex in the above measurements does not differ significantly between strains for either S1 or V1 across ages (Figure 4D,H), suggesting that the changes in cortical thickness are due to a generalised increase or decrease in all cortical layers.

### Altered cortical size in BTBR mice may be due to a change in global cell number, rather than changes in specific neuronal populations or apoptotic pathways

**Figure 5:**
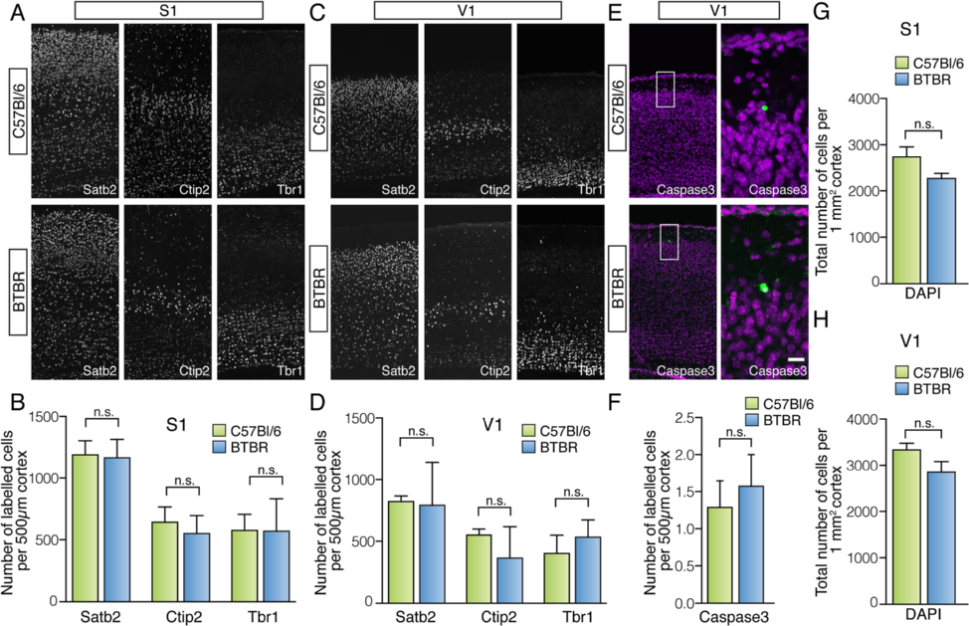
**Quantification of neuronal number in specific cortical populations, apoptosis and cell density during development in C57Bl/6 and BTBR mice.** Coronal sections of P7 C57Bl/6 and BTBR brains were processed for immunohistochemistry using anti-Satb2, anti-Ctip2 and anti-Tbr1 antibodies to label neurons in different cortical layers. Satb2 is highly expressed in layers 2/3 and less highly expressed in deep layers; Ctip2 is highly expressed in neurons in layer 5 and weakly expressed in layer 6, and Tbr1 is highly expressed in layer 6 **(A, C)**. The expression patterns of Ctip2, Tbr1 and Satb2 are indistinguishable in both S1 and V1 between mouse strains, indicating normal cortical neuronal distribution and normal cortical lamination in BTBR mice. This was confirmed by analysis of the number of labelled cells per 500 μm of cortex, which was found to be unchanged between strains in both S1 and V1 **(B, D)**. Caspase3 staining revealed that the degree of apoptotic cell death (quantified by number of labelled cells, green, in a 500-μm segment of DAPI-stained (purple) V1 cortex) was not significantly different between strains at this age **(E, F)**. The density of DAPI-stained cells per 1 mm^2^ of cortex was found to be no different between C57Bl/6 and BTBR mice in S1 **(G)** and V1 **(H)**. *n* = 7 animals for each strain and condition. Scale bar = 100 μm for (A), (C) and (E); 20 μm for (E) insert. Ctip2 = chicken ovalbumin upstream promoter transcription factor-interacting proteins 2, Tbr1 = T-box brain gene 1, Satb2 = special AT-rich sequence-binding protein 2, BTBR = BTBR T + tf/J, DAPI = 4′,6-diamidino-2-phenylindole, dihydrochloride, n.s. = not significant.

To further investigate the cellular processes underlying these developmental changes in BTBR cortical size, we then assessed the laminar organisation present within S1 and V1 during development. Both strains were analysed with immunohistochemistry for three different cortical layer markers at P7 (Figure 5A,B,C,D; *n* = 7 for each strain). These markers were as follows: special AT-rich sequence-binding protein 2 (Satb2), which is expressed by cortical neurons in layers 2/3 and is essential for the development of callosal projections [42-44]; chicken ovalbumin upstream promoter transcription factor-interacting protein 2 (Ctip2), which is a marker of subcortically projecting neurons in layer 5 [45,46]; and T-box brain gene 1 (Tbr1) which is expressed in early-born glutamatergic cortical neurons in layer 6 and is important for the formation of the subplate and layer 6 [47]. We found that the distribution patterns of neuronal populations positive for Satb2, Ctip2 and Tbr1 in both S1 and V1 are similar in BTBR and control animals at P7 (Figure 5A,C), indicating that cortical lamination at a gross level is not disrupted in BTBR mice. Furthermore, the total number of labelled cells per 500 μm of cortex is not significantly different between strains within either S1 or V1 (Figure 5B,D; Additional file 3; *n* = 7 for each strain). This indicates that the changes in cortical thickness exhibited by BTBR animals are not due to an increase in cell number of a specific cortical laminar population. Alternatively, a change in cortical thickness could also be due to differences in cellular apoptosis between strains. To investigate this, the average number of Caspase3-labelled cells within 500 μm of developing cortex was quantified (*n* = 7 for each strain). This revealed that the number of Caspase3-positive cells (green in Figure 5E) is unchanged between the two strains at P7 (Figure 5E,F; Additional file 3), indicating that developmental changes in cortical thickness are not due to alterations in apoptotic pathways. Finally, in order to determine whether the change in cortical thickness of BTBR brains is due to a change in cortical cell density, rather than a general increase in cell number, we quantified the density of DAPI-stained nuclei per 1 mm^2^ of cortex (*n* = 7 animals for each strain). Despite the increase in cortical thickness in V1 of BTBR animals at P7, we did not find a significant difference in cell density between the two strains in either S1 (Figure 5G) or V1 (Figure 5H; Additional file 3), indicating that the enlargement of BTBR V1 is likely due to an increase in overall cell number. We did, however, observe a non-significant trend towards a lower density of cells in the BTBR strain, potentially indicating that factors other than overall cell number may contribute to the changes in cortical size throughout development, such as changes in intracortical connectivity.

Together, the above results suggest that the change in cortical thickness in BTBR animals occurs without defects in lamination and does not involve changes in neuronal number of specific cortical populations, or alterations in apoptosis, as has previously been suggested in human ASD patients [48]. Rather, it is likely that these changes occur at least partially via a global change in cell number, perhaps via proliferation and/or migration pathways, ultimately maintaining cortical density despite changes in cortical size.

## Discussion

Significant increases in the rate of ASD diagnosis and the cost of treatment over the lifespan of these patients have prompted further research to discover better diagnostic and therapeutic strategies for this disorder. To further this goal, animal models have been developed to investigate the anatomical and functional abnormalities displayed in autistic brains [4,19,49]. The BTBR mouse is an inbred strain that is currently used as a model of autism [19,49]. Previous neuroimaging studies have highlighted malformations of white matter connections in this strain [16,17,21]. Among these findings, complete absence of the corpus callosum and a significantly reduced hippocampal commissure have been reported [22], as well as ectopic interhemispheric connections [16,21]. However, whether BTBR mice also display disruptions in the formation of functional areas within the cortex is not known. Here, we found that BTBR mice do not exhibit any interhemispheric neocortical connectivity through either normally or ectopically occurring commissures. We also revealed that the positions of both S1 and V1 are shifted medially and that there are age- and cortical-area-dependent changes in cortical thickness. These results suggest that significant alterations in cortical area patterning and connectivity are present in the BTBR mouse strain that may relate to previously observed sensory or behavioural deficits (for example in social interaction) [14,19].

In marsupials, which lack a corpus callosum, neocortical axons connect interhemispheric areas via the anterior commissure (reviewed in [50]). Similarly, in select patients with complete agenesis of the corpus callosum, it has been suggested that some neocortical axons re-route via the anterior commissure [51]. In the BALB/cCF mouse strain, in which some animals fail to develop a corpus callosum, the anterior commissure is not enlarged, but the density of axons projecting through this commissure increases, possibly compensating for the loss of callosal axons [52,53]. We confirmed that BTBR mice have complete agenesis of the corpus callosum, form longitudinal Probst bundles on either side of the midline and have reduced hippocampal and anterior commissure volume. Similarly, we showed that the anterior and hippocampal commissures are unlikely to compensate for the loss of callosal connections between cortical areas in BTBR mice. This result is particularly interesting in light of a recent study showing that cortical areas can be connected via ectopic projections in the anterior and posterior commissures in acallosal humans [54]. Furthermore, patients with agenesis of the corpus callosum and no anterior commissure display only subtle defects in tests requiring interhemispheric communication [51], indicating that other mechanisms may be involved in such functional compensation.

Areal patterning of the neocortex is a critical event in mammalian brain development. Alterations in the shape and size of one primary cortical area can cause changes in other spatially related areas in the neocortex [28], which could eventually have dramatic effects on brain function, particularly perception and behaviour in adult animals [55-57]. Over the past decade, molecular manipulation of the cortex has demonstrated that neocortical area patterning is initiated by genetic regulation intrinsic to the neocortex and is later refined and maintained by peripheral activity transmitted via thalamocortical axons (extrinsic factors; [28], [58-60]). The correct formation of the barrel field in the BTBR cortex suggests that the gross formation of thalamocortical circuitry occurs normally in BTBR animals, as sensory afferents accurately target their correct cortical layer and form characteristic barrel patterns. However, whether the fine topography of this system is maintained remains to be investigated.

Our data demonstrate a significant shift in the position of both S1 and V1 towards the midline, together with a significant reduction in the size of V1 in BTBR mice compared with C57Bl/6 mice. Considering the similar size of the neocortex in these two strains at P10, the medial shift of cortical areas could be due to a direct effect of changes in the expression of patterning genes or an indirect effect caused by the simultaneous shrinkage of the medial cortex and expansion of the lateral cortex or changes in white matter organisation. Whatever the causal factor/s, it is tempting to speculate that the observed medial shift in cortical area position in BTBR mice may impact overall cortical function if the space available for adjacent cortical areas associated with higher level processing is reduced. The observed significant reduction in the size of V1 occurs consistently across different stages of development. Further experiments are required to determine if BTBR mice demonstrate deficits in vision as a result of this defect.

Normal cortical lamination in the adult somatosensory cortex has been reported in other strains of acallosal mice, as well as adult mice that have undergone postnatal midline transection [35,36]. These studies also revealed altered cortical thickness in these mice, in association with comparatively normal neuronal density in neocortical regions that usually have abundant callosal connections. Although adult cortical thickness does not significantly differ between the two strains, we observed a trend towards a thinner cortex in the BTBR mouse, a result that is in agreement with other data showing a widespread decrease in adult cortical thickness in this strain measured by structural MRI [16]. However, we have made the additional finding that a different trend is present during development. At P7, BTBR mice display an increase in cortical thickness in V1, but no change in S1. Similar to the findings in other acallosal models [35,36], we also found that the relative thickness of layers as well as the laminar neuronal populations and degree of apoptosis remains intact despite these alterations. Despite the change in cortical thickness, the total cell density in the cortex is also unchanged between strains, showing that a subtler, global change in the neurogenesis and the migration of newly born neurons in BTBR mice may underlie the observed changes [40]. However, as there is a general trend towards reduced density in the BTBR brains, it is possible that other mechanisms also contribute to changes in cortical thickness. For example, alterations in the intracortical connectivity of BTBR brains (such as a change in the size of dendritic arbours, possibly in response to a loss of interhemispheric connections) may also affect the cortical thickness in this strain.

These results showing changes in the formation of cortical areas in a mouse model of ASD are of potential significance for understanding the basis of this disorder in humans. Recent studies comparing gene expression patterns between ASD patients and controls have shown that the region-specific expression patterns of genes that typically distinguish frontal and temporal cortices, but not the occipital and cerebellar regions, are significantly attenuated in autistic brains [61,62]. Moreover, a haplotype analysis of 393 ASD patients in Han Chinese revealed that a haplotype of Wnt2 (rs2896218-rs6950765: G-G), a central nervous system patterning gene, is significantly associated with ASD [63]. Thus, cortical area patterning may be disrupted in ASD patients and may contribute to their aberrant behavioural phenotypes.

One of the most interesting findings of this study involves the variable changes in BTBR animals throughout development. Both the cortical thickness and arealisation analyses revealed a general trend towards larger values in the BTBR strain during development that was then reversed in adulthood. Interestingly, ASD patients also exhibit these changes during development, such as transient childhood increases in brain size and cortical thickness [64,65]. These changes are not global, but rather vary between different cortical areas, similar to our observations in BTBR animals [65]. The mechanisms underlying this characteristic of ASD as well as its functional implications remain unclear. Future studies could investigate this aspect of brain development in the BTBR strain as a model for understanding the aetiology and plasticity of ASD more broadly.

## Conclusions

Here, we provide the first evidence that the size, position and thickness of cortical areas are disrupted in the BTBR model of ASD and that these changes occur in varied ways throughout the lifespan of the animal. Using immunohistochemical analyses, we show that these changes are likely due to developmental alterations in total cell number within discrete cortical areas, rather than changes in specific neuronal populations or apoptotic pathways. These anatomical deficits share similarities with trends observed in human ASD patients and may account for some of the autistic-like behavioural abnormalities observed in BTBR mice.

## Methods

### Animals

The BTBR (stock number 002282) line and the C57Bl/6 (stock number 000664) line were both originally sourced from the Jackson Laboratory. All mouse strains were bred at The University of Queensland. Both breeding and experimental protocols were approved by the University of Queensland Animal Ethics Committee and were performed according to the Australian Code of Practice for the Care and Use of Animals for Scientific Purposes. Both female and male mice were used for all adult and postnatal experiments.

### Tissue preparation

For postnatal pups or adult animals, 185 mg/kg sodium pentobarbitone was injected intraperitoneally. Animals were then transcardially perfused with 0.9% saline solution (0.9% *w*/*v* NaCl in MilliQ^TM^ water; Millipore, Billerica, USA), followed by freshly prepared 4% *w*/*v* paraformaldehyde (PFA; ProSciTech, Thuringowa Central, Australia) in phosphate-buffered saline (PBS; pH 7.4; Lonza, Basel, Switzerland). The head was removed and post-fixed at 4 °C in 4% PFA in PBS until required for tissue processing. For tangential sections, animals were transcardially perfused with 0.9% saline solution to remove the blood from the brain. The cortices were then dissected and flattened between two slides approximately 1 mm apart and fixed in 4% PFA in PBS at 4 °C for at least 48 h. Following fixation, the flattened cortices were transferred into PBS and maintained at 4 °C until required for sectioning. Prior to sectioning, brains were blocked in 3% *w*/*v* Difco™ Noble agar (Becton, Dickinson and Company, Franklin Lakes, USA) in MilliQ water. Free-floating sections of 50-μm thickness were cut using a vibratome (Leica Biosystems, Jurong, Singapore).

### MRI data acquisition

*Ex vivo* MRI data were acquired using a 16.4 Tesla vertical bore, small animal MRI system (ParaVision v5.0; Bruker Biospin, Madison, USA) and a 15-mm linear, surface acoustic wave coil (M2M Imaging, Brisbane, Australia). Brain samples were washed in PBS 4 days prior to scanning and placed in Y06/06 perfluoroether Fomblin oil (Solvay Solexis, Brussels, Belgium). High-resolution *T*_1_-weighted anatomical images were acquired using a three-dimensional (3D) fast low-angle shot (FLASH) gradient echo sequence at 50-μm isotropic resolution, using TR/TE = 50/12 ms, flip angle 30° and 2 averages, with an acquisition time of 1 h. High angular resolution diffusion-weighted imaging (HARDI) was acquired using 3D diffusion-weighted spin-echo images as previously described [66] at 100-μm isotropic resolution. Each dataset was composed of two *b*0 values (*b* value of 0 and 5,000 s/mm^2^, *δ*/*∆* = 2.5/14 ms), 30 diffusion-weighted images, 1 average and 1.5 partial-Fourier acceleration factors in the phase dimensions, with an acquisition time of 15 h.

### Probabilistic fibre tracking and volumetric measurements

The HARDI data were processed using the Constrained Spherical Deconvolution (CSD) model (MRTrix 0.2.9) with *l*_max_ = 6. Fibre tract streamlines were generated using probabilistic tracking with step size 0.01 and curvature 0.04. To visualise the morphology of the whole brain commissural tracts, 100,000 streamlines were generated in the mid-lateral direction using the mid-sagittal plane as the seeding point. To quantify the major commissural fibre tracts, 10,000 streamlines were generated for each of the anterior commissure, the hippocampal commissure and the corpus callosum from ROIs identified in the mid-sagittal plane through the cortical midline of the fractional anisotropy colour maps.

Volumetric measurements of brain structures were performed by registration of a segmented *ex vivo* adult C57Bl/6 MRI atlas [67] to the anatomical images using the FSL5.0 non-linear registration program (fsl.fmrib.ox.ac.uk/). Results were statistically compared using Student’s *t*-tests.

### Immunohistochemistry and quantification of cell number

Free-floating sections were incubated for 2 h in 0.9% *v*/*v* hydrogen peroxide in blocking solution: 2% *v*/*v* normal goat serum (Vector Laboratories, Burlingame, USA) or normal donkey serum (Jackson Laboratories, Bar Harbor, USA) and 0.2% *v*/*v* Triton-X 100 (Sigma-Aldrich, St. Louis, USA) in PBS. Primary antibodies including rat anti-Ctip2 monoclonal antibody (1:500; Abcam, Cambridge, UK), rabbit anti-Tbr1 polyclonal antibody (1:500; Santa Cruz Biotechnology, Inc., Dallas, USA), rabbit anti-Satb2 polyclonal antibody (1:500; Abcam, Cambridge, UK), rabbit anti v-Glut2 polyclonal antibody (1:500; Synaptic Systems, Goettingen, Germany) and rabbit anti-cleaved Caspase3 antibody (1:500; Cell Signaling, Danvers, USA) were diluted in blocking solution and applied overnight at room temperature. After 3 × 20-min washes with PBS, the sections were incubated with the secondary antibody diluted in 0.2% *v*/*v* Triton-X 100 in PBS for 1 h. The secondary antibodies used were biotinylated donkey-anti-rabbit IgG (1:500; Vector Laboratories, Burlingame, USA) and biotinylated donkey-anti-rat IgG (1:500; Jackson Laboratories, Bar Harbor, USA). The sections were washed with PBS for 3 × 20 min and then stained with Alexa Fluor 647-conjugated Strepavidin (Invitrogen, Waltham, USA). Where amplification was not needed, donkey-anti-rabbit Alexa Fluor 488 (1:500; Abcam, Cambridge, UK) secondary antibody was applied for 3 h. After 3 × 10-min washes with PBS, the sections were counterstained with DAPI and coverslipped using Prolong gold antifade reagent (Invitrogen, Waltham, USA). After imaging, 500-μm-wide regions of the complete neocortex were analysed for the number of positively stained cells. This number was then divided by the area of cortex in which DAPI-stained cells were counted and then multiplied by 1 million to quantify the cellular density as number of cells per 1 mm^2^ cortex. All values were statistically compared between conditions by Student’s *t*-tests or Mann-Whitney tests (*n* = 7 animals for each strain in all conditions).

### Image acquisition

Brightfield imaging was performed with a Zeiss upright Axio-Imager Z1 microscope fitted with an Axio-Cam HRc camera. Confocal fluorescent images were acquired as single 0.6-μm-thick optical sections using a Zeiss inverted Axio-Observer fitted with a W1 Yokogawa spinning disk module and Hamamatsu Flash4.0 sCMOS camera and Slidebook 5.5 software. Images were pseudocoloured to permit overlay and then were cropped, sized and contrast-brightness enhanced for presentation with Adobe Photoshop software.

### Quantification of cortical thickness in BTBR and C57Bl/6 mice

DAPI or haematoxylin staining was performed on 50-μm coronal sections from adult BTBR and C57Bl/6 mouse brains (*n* = 6 for each strain; equal numbers of DAPI or haematoxylin staining for each condition) to reveal gross cortical anatomy. For haematoxylin staining, sections were first sequentially mounted onto gelatin-coated SuperFrostPlus slides (Menzel-Gläser, Brunswick, Germany) and allowed to air dry for approximately 30 min. Sections were then hydrated with PBS and bathed in Mayer’s haematoxylin (Sigma-Aldrich, St. Louis, USA) for 5 min. The reaction was then stopped by rinsing the sections in tap water for 5 min, after which they were immersed sequentially in a series of graded ethanols, followed by xylene, then coverslipped with DPX mounting medium. Following imaging of the slides, measurements of cortical thickness and layer size were conducted in the centre of S1 and V1 in comparable coronal sections from each animal of both mouse strains. Quantification at P7 was performed on DAPI-stained sections resulting from the previously described immunohistochemical staining (*n* = 7 for each strain). For layer proportion analysis, the absolute thickness of each layer was converted into a proportion of the total cortical thickness for that single brain and then each layer was statistically compared between strains. Results were statistically compared with Student’s *t*-tests.

### Quantification of relative size and position of cortical areas in BTBR and C57Bl/6 mice

Tangential sections of P7, P10 and P22 cortices (stained for v-Glut2) of both mouse strains were imaged and then unbiased measurements were obtained from de-identified images for strain. Following analysis, images were then reassigned their strain genotype based on a numerical code. The PMBSF, V1 and the whole neocortex in both hemispheres in C57Bl/6 and BTBR mice were manually outlined using ImageJ (National Institutes of Health). We defined the longest diameter of the flattened neocortex (distinguished from the olfactory cortex by the bordering rhinal fissure) as the ‘length’ of the neocortex, and the ‘width’ was measured on the axis perpendicular to this. Next, the areas of the PMBSF, V1 and the whole neocortex were measured. The third barrel in row C (the C3 barrel) was then used as a central point for the PMBSF in the analysis of the relative position of the PMBSF, while the apex of the v-Glut2-stained area of V1 was used to identify the relative position of V1. Using these points, we measured the anterior and medial positions of the centre of the C3 barrel and the position of the most rostral tip of V1. The relative positions of the PMBSF and V1 were calculated by normalising their anterior length or medial length according to the total length or width of the neocortex, respectively. All results were then compared between strains for each age using a Student’s *t*-test.

## Additional files

Additional file 1: Table S1. Cortical area measurements as graphed in Figure 3. Table of mean, SEM and *P* values for the graphical data in Figure 3. Additional file 2: Table S2. Cortical thickness and layer proportion as graphed in Figure 4. Table of mean, SEM and *P* values for the graphical data in Figure 4. Additional file 3: Table S3. Transcription factor, Caspase3 and DAPI cell counts as graphed in Figure 5. Table of mean, SEM and *P* values for the graphical data in Figure 5.

## Abbreviations

3D: three-dimensional
ASD: autism spectrum disorder
BTBR: BTBR T + tj/J
CSD: Constrained Spherical Deconvolution
Ctip2: chicken ovalbumin upstream promoter transcription factor-interacting proteins 2
DAPI: 4′,6-diamidino-2-phenylindole, dihydrochloride
FLASH: fast low-angle shot
HARDI: high angular resolution diffusion-weighted imaging
MRI: magnetic resonance imaging
PBS: phosphate-buffered saline
PFA: paraformaldehyde
PMBSF: posterior medial barrel subfield
ROI: region of interest
S1: primary somatosensory cortex
Satb2: special AT-rich sequence-binding protein 2
Tbr1: T-box brain gene 1
V1: primary visual cortex
v-Glut2: vesicular-glutamate transporter 2
Wnt2: wingless-type MMTV integration site family, member 2
